# Self-reported health and life satisfaction in older emergency department patients: sociodemographic, disease-related and care-specific associated factors

**DOI:** 10.1186/s12889-021-11439-8

**Published:** 2021-07-21

**Authors:** Anna Schneider, Dorothee Riedlinger, Mareen Pigorsch, Felix Holzinger, Johannes Deutschbein, Thomas Keil, Martin Möckel, Liane Schenk

**Affiliations:** 1 Charité – Universitätsmedizin Berlin, corporate Member of Freie Universität Berlin and Humboldt-Universität zu Berlin, Institute of Medical Sociology and Rehabilitation Science, Berlin, Germany; 2 Charité – Universitätsmedizin Berlin, corporate Member of Freie Universität Berlin and Humboldt-Universität zu Berlin, Department of Emergency Medicine, Campus Virchow-Klinikum and Campus Charité Mitte, Berlin, Germany; 3 Charité – Universitätsmedizin Berlin, corporate Member of Freie Universität Berlin and Humboldt-Universität zu Berlin, Institute of Biometry and Clinical Epidemiology, Berlin, Germany; 4 Charité – Universitätsmedizin Berlin, corporate Member of Freie Universität Berlin and Humboldt-Universität zu Berlin, Institute of General Practice, Berlin, Germany; 5 Charité – Universitätsmedizin Berlin, corporate Member of Freie Universität Berlin and Humboldt-Universität zu Berlin, Institute of Social Medicine, Epidemiology and Health Economics, Berlin, Germany; 6 University of Wuerzburg, Institute of Clinical Epidemiology and Biometry, Wuerzburg, Germany; 7 State Institute of Health, Bavarian Health and Food Safety Authority, Bad Kissingen, Germany

**Keywords:** Emergency department, Self-reported health, Life satisfaction, Sociodemographic factors, Vulnerable populations

## Abstract

**Background:**

Self-reported health (SRH) and life satisfaction (LS) are patient-reported outcomes (PROs) that independently predict mortality and morbidity in older adults. Emergency department (ED) visits due to serious health problems or accidents might pose critical life events for patients. This study aimed (a) to characterize older patients’ SRH and LS during the distinct event of an ED stay, and (b) to analyze concomitant associations of PROs with ED patients’ sociodemographic, disease-specific and care-related variables.

**Methods:**

Study personnel recruited mostly older ED patients from three disease groups during a two-year period (2017–2019) in eight EDs in central Berlin, Germany, in the context of the health services research network EMANet. Cross-sectional data from the baseline patient survey and associated secondary data from hospital information systems were analyzed. Multilevel linear regression models with random intercept were applied to assess concomitant associations with SRH (scale: 0 (worst) to 100 (best)) and LS (scale: 0 (not at all satisfied) to 10 (completely satisfied)) as outcomes, including sensitivity analyses.

**Results:**

The final sample comprised *N* = 1435 participants. Mean age was 65.18 (SD: 16.72) and 50.9% were male. Mean ratings of SRH were 50.10 (SD: 23.62) while mean LS scores amounted to 7.15 (SD: 2.50). Better SRH and higher LS were found in patients with cardiac symptoms (SRH: β = 4.35, *p* = .036; LS: β = 0.53, *p* = .006). Worse SRH and lower LS were associated with being in need of nursing care (SRH: β = − 7.52, *p* < .001; LS: β = − 0.59, *p* = .003) and being unemployed (SRH: β = − 8.54, *p* = .002; LS: β = − 1.27, *p* < .001). Sex, age, number of close social contacts, and hospital stays in the previous 6 months were additionally related to the outcomes. Sensitivity analyses largely supported results of the main sample.

**Conclusions:**

SRH and LS were associated with different sociodemographic and disease-related variables in older ED patients. Nursing care dependency and unemployment emerged as significant factors relating to both outcomes. Being able to identify especially vulnerable patients in the ED setting might facilitate patient-centered care and prevent negative health outcomes. However, further longitudinal research needs to analyze trajectories in both outcomes and suitable intervention possibilities in the ED setting.

**Trial registration:**

EMANet sub-studies were registered separately: German Clinical Trials Register (EMAAge: DRKS00014273, registration date: May 16, 2018; https://www.drks.de/drks_web/navigate.do?navigationId=trial.HTML&TRIAL_ID=DRKS00014273; EMACROSS: DRKS00011930, registration date: April 25, 2017; https://www.drks.de/drks_web/navigate.do?navigationId=trial.HTML&TRIAL_ID=DRKS00011930); ClinicalTrials.gov (EMASPOT: NCT03188861, registration date: June 16, 2017; https://clinicaltrials.gov/ct2/show/NCT03188861?term=NCT03188861&draw=2&rank=1).

**Supplementary Information:**

The online version contains supplementary material available at 10.1186/s12889-021-11439-8.

## Background

Emergency departments (EDs) in most Western countries face an increase in presentations by older persons with complex health care needs, e.g., multimorbid patients and those with unmet psychosocial needs in combination with somatic complaints [1, 2]. EDs are distinctive care and research settings at the interface of ambulatory and inpatient health care. Time-sensitive patient presentations and high staff workload render EDs a challenging environment for clinicians [3] and patients alike [4]. Serious health problems or accidents might pose critical life events for patients with potentially considerable implications for their psychological and physical well-being during hospitalization and after discharge [5–7]. Currently, most health care services with their ‘silo’ structures are not designed for the delivery of comprehensive care which considers all of the patients’ needs in one setting [8–10]. Although initiatives at addressing a variety of patients’ health and psychosocial needs in the health care setting exist (e.g. [11]), crowded ED environments and lack of respective clinician training in identifying and handling patients with complex needs further impedes adequate responses [12].

Diminished self-reported health was found to be a predictor of higher mortality risk, even after adjusting for objective disease markers [13, 14]. To date, research on patients’ subjective well-being in the ED setting mainly focused on screening for comorbid psychological distress in patients presenting with somatic symptoms [5] or patients’ affective states during their ED stay [15]. These studies identified sociodemographic factors and social determinants, such as sex, marital status, age and education, as well as illness-related variables, such as triage category and comorbidities, as risk factors for psychological distress in ED patients [5]. Self-reported health was closely linked to psychological distress [5, 16]. More tapered research investigated self-reported health in the ED setting as one of multiple outcomes in diverse samples of ED populations (e.g. [11, 17–19]. However, studies analyzing concomitant associations of ED patients’ sociodemographic, disease-specific and care-related variables with self-reported health as a primary outcome are lacking. The rationale for inquiring self-reported health lies, inter alia, in its predictive value for patient mortality and its potential use for early identification of especially vulnerable patients in clinical settings [14].

Furthermore, life satisfaction has emerged as a potentially important predictor of mortality and morbidity, although research results on respective associations were mixed [20, 20]. However, studies on patients’ life satisfaction scores in the ED setting are missing. Generally, individuals’ health and life satisfaction were positively associated suggesting that higher well-being comes with better health and vice versa [20, 21]. Furthermore, higher life satisfaction was associated with fewer chronic health conditions and lower mortality rates, although the latter association was significantly alleviated by participants’ health status [22, 23]. Life satisfaction is a distinct dimension of psychological well-being [23, 24] and represents an individual’s cognitive assessment of overall satisfaction with different life aspects [25]. In case of global life satisfaction scales, research showed that people tend to evaluate life components that are important to them and are rather stable over time, e.g., one’s own status concerning work, relationships, or health [26]. However, life satisfaction is also determined by factors like genetic predisposition, personality traits, occurrence of specific life events, cultural background [26], as well as sociodemographic variables and social determinants, i.e., sex, age, educational level, marital status, net personal income and work-related factors [27, 28].

The study’s first aim was to characterize the self-reported health and life satisfaction during a distinct event, an ED visit, in a sample of mostly older patients from three different disease groups in a multi-center study located in Germany. In its second aim, this study further analyzed associations between sociodemographic as well as disease-specific and care-related factors with self-reported health and life satisfaction in ED patients considering the multidimensionality of both outcomes.

## Methods

### Study design and setting

This study used data collected in the context of EMANet, which is the Emergency and Acute Medicine Network for Health Care Research in Berlin. In its first funding phase from 2016 to 2020, network institutions employed a mixed-methods study design aiming at the analysis of characteristics and health services use patterns of mostly older multimorbid ED patients with ambulatory care sensitive conditions. Applied research methods included a multi-center prospective cohort study of three distinct patient samples, qualitative interviews with ED patients and ED staff, as well as secondary data analysis of ED patients’ hospital records [33]. The present study was based on the analysis of cross-sectional data from the baseline survey of the EMANet cohort and linked participant data from electronic patient records. EMANet sub-studies included EMASPOT, which addressed ED patients with cardiac symptoms and potentially co-morbid mental health disorders; EMACROSS, which targeted ED patients with respiratory complaints; and EMAAge, which investigated ED patients with proximal femoral fractures. These three patient samples and their respective disease groups stood exemplary for a considerable share of ED presentations requiring subsequent hospital admission in Germany, i.e., patients with cardiovascular diseases, respiratory diseases and hip injuries [1]. Participants were recruited from eight EDs in Berlin’s central district Mitte. Recruiting EDs are located in hospitals with differing annual ED patient volumes (ranging between 10,000 and 110,000 adult patients per year), inpatient bed capacities (ranging between 150 and 1300 beds), and ownership (public or non-profit). Two recruiting EDs belong to a university hospital. Initial sample size calculations for each sub-study were based on study-specific primary research questions and prevalence estimates of patient populations with respective symptoms and diagnoses in participating EDs. No post-hoc sample size calculations were conducted for this data analysis. Each of the three sub-studies was approved by the ethics committee of the Charité - Universitätsmedizin Berlin (EMAAge: EA1/362/16; EMACROSS: EA1/361/16; EMASPOT: EA1/363/16). In Germany, access to the ED is not formally regulated. Practitioners can refer patients to EDs and patients can refer themselves to an ED of their choice. Apart from walk-in patients, transport to the ED can occur by non-urgent medically accompanied patient transport (mostly for chronically ill and mobility-impaired patients) or by emergency medical services (EMS), i.e., accompanied by paramedics or emergency physicians. Both types transport patients to the next suitable ED after a respective request [29].

### Study recruitment

Trained study personnel, not involved in regular patient care, recruited patients between June 1, 2017 and June 28, 2019. The first step in the patient screening process included the verification of a patient’s presenting symptoms and ICD-10 diagnosis according to inclusion criteria of respective sub-studies (see Supplementary Table 1). Study nurses evaluated eligibility by monitoring hospital information systems on patients concurrently treated in participating EDs during fixed time periods. If patients met inclusion criteria, study nurses approached them to verify eligibility. The screening process was documented in printed structured questionnaires and manually transferred into an electronic database on a daily basis. For eligible patients who refused to participate or who could not be reached, sex, age and a non-response reason were gathered. Once patients gave informed consent, study nurses conducted an interview of approximately 30 to 60 minutes with handheld tablets in ED premises. In EMAAge, legal guardians or patients’ relatives were included if patients were not able to give informed consent. Eligible EMAAge patients or respective proxies were surveyed up to 7 days after ED treatment and hip surgery, i.e., mostly during their subsequent hospital stay. Printed study materials were additionally available in four languages: German, English, Arabic, and Turkish. All participants gave written permission for review of their individual electronic health records for study-specific aims.

### Measures

For all three study populations a shared set of questions was surveyed. Patients’ sociodemographic, disease-specific and care-related factors were obtained from surveys and medical records. In surveys, patients reported sociodemographic information, i.e., sex, birth date, own and parents’ countries of birth, educational background, and current employment status. Furthermore, the number of close social contacts (defined as persons that participants could rely on in case of severe personal problems), care dependency, and health care services use in the previous 6 months (including general practitioner (GP) visits, ED visits and hospital stays) were retrieved. Response formats for these items included pre-defined categories, while free-text responses comprised specifications of country of birth, type of vocational qualification, and type of employment. For data analyses, the migration background of study participants was coded into a) no migration background (participants and both parents born in Germany) and b) migration background (participant not born in Germany or participant born in Germany with at least one parent born outside of Germany). Educational attainment was operationalized by using data on the educational and vocational qualification of participants based on the procedure recommended by the Comparative Analysis of Social Mobility in Industrial Nations (CASMIN) classification of education: primary level (up to general elementary education and vocational qualification), secondary level (up to general maturity certificate and vocational qualification), and tertiary level (up to higher tertiary education) [30]. Additional to survey measures, the following information was retrieved from participants’ medical records in hospital information systems: Manchester Triage System (MTS) category at the time of ED presentation, subsequently coded into urgent (MTS level 1 to MTS level 3) and non-urgent (MTS level 4 and MTS level 5); case type (discharged from ED or admitted as inpatient); and transport to the ED, subsequently coded into walk-in (patient presented individually at ED), transport by ambulance services, transport by emergency medical services (EMS), and EMS transport accompanied by an emergency physician. We classified independent variables into sociodemographic factors (i.e., age, sex, education, employment status, migration background, social contacts), disease-specific factors (sub-study, care dependency, case type, MTS level, transportation to ED) and care-related factors (GP visit, ED visit, hospital stay).

Self-reported health was measured with the visual analogue scale (VAS) from the EQ-5D instrument [31]. Study participants were asked to indicate their overall health on the day of the interview on a vertical scale from 0 (*worst imaginable health*) to 100 (*best imaginable health*). Life satisfaction was measured with one item inquiring participants to rate their current overall satisfaction with their life on a scale ranging from 0 (*not at all satisfied*) to 10 (*completely satisfied*) [32]. The application of generic as opposed to illness-specific measures of health and life satisfaction enables the comparison of outcomes across different disease groups [34].

### Statistical analysis

Descriptive statistics depict absolute and relative frequencies for categorical variables as well as means and standard deviations for continuous variables. In non-responder analysis, participants and those who declined to participate were compared with regard to their age (t-test for independent samples) and sex (Pearson’s chi-square test). For the analysis of bivariate associations between variables, a standardized chi-square metric (contingency coefficient Cramer’s V) was applied for associations between nominal variables, the Eta coefficient η^2^ for associations between nominal and metric variables and, Pearson’s r for associations between metric variables. Cut-off points for the classification of the strength of correlations were grouped into low (correlation coefficient < 0.35), moderate (correlation coefficient between 0.36 and 0.67), and high (correlation coefficient > 0.68) [33].

No data was imputed in case of missing values. Due to the explorative character of this study, we did not adjust for multiple testing [35]. Independent variables for multivariable analyses were selected based on the above described previous research results on associated factors of self-reported health and life satisfaction as well as substantive considerations of authors regarding further potentially relevant disease-specific and care-related factors for respective outcomes in ED patients. We decided to apply multilevel analysis in order to consider potential differences in outcomes due to the considerable structural heterogeneity of study EDs regarding patient volumes/ ED size, hospitals’ inpatient bed capacities and hospital ownership, as well as potential underlying differences in the composition of the patient population and case-mix of study EDs. To account for clustering of data on this higher level of recruiting EDs, multivariable procedures included multilevel linear regression models with random intercept (first level: participant data, second level: EDs). Estimates of regression coefficients from multilevel linear models include 95% confidence intervals (CI). For indication of the quality of model fit, i.e., amount of variance explained by fixed effects and random effects, we report marginal R^2^, conditional R^2^ and the intraclass correlation coefficient (ICC) for each statistical model. Descriptive and bivariate statistical analyses were conducted with SPSS V25.0 (IBM Inc., Chicago, USA). Multilevel linear regression models were calculated with R version 4.0.2, packages lme4 and MuMIn [36, 37]. For main analyses, the full dataset with information from all three sub-studies (i.e., EMAAge, EMACROSS, and EMASPOT) was used. Additional sensitivity analyses were applied with regard to the impact of the inclusion of participants who were interviewed after their ED stay (scenario B) and participants of younger age (scenario C). We hypothesized that both factors, i.e., being interviewed outside of the ED and being of younger age, might have influenced the reliability of patient reports regarding outcome variables. We thus ran multilevel linear regression models as sensitivity analyses with identical variables with data samples excluding EMAAge participants (scenario B) and excluding all participants under the age of 50 years from the full dataset of all sub-studies (scenario C).

## Results

### Study population

**Fig. 1:**
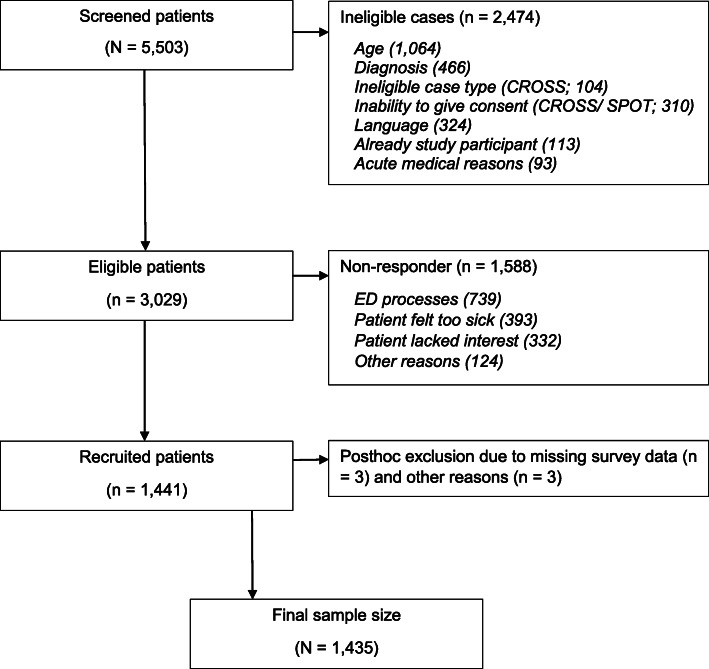
Flow chart of patient recruitment in EMANet

Overall, study personnel screened *N* = 5503 ED patients (see Fig. 1). Following inclusion and exclusion criteria, 2474 patients (45.0%) were excluded in the screening process with the main reasons for exclusion being inappropriate age (*n* = 1064) and inappropriate ED diagnosis (*n* = 466). In this first step, excluded patients were counted as neutral non-responders since their ineligibility for study participation according to inclusion criteria of the three sub-studies did not affect the final size of the eligible study population. Of the remaining 3029 eligible patients in the second step, *n* = 1588 patients (52.4%) refused to participate or could not be recruited and counted as non-responders. Main reasons for non-participation in the second step were research-practical reasons and ED processes which prevented the approach of eligible patients (*n* = 739), e.g., missing research infrastructure in study EDs or closely timed diagnostic and treatment processes of ED patients.^1^ Statistical analysis indicated that non-responders tended to be older (mean age of non-responders = 66.28 years vs. mean age of participants = 65.20 years, *p* = .080) but did not differ with regard to sex (number of female non-responders = 766 vs. number of female responders = 707, *p* = .662) compared to study participants.^2^ Post-hoc, six patients were excluded due to missing survey data or due to ineligible final diagnosis. The final sample thus comprised *N* = 1435 participants (EMAAge: *n* = 322; EMACROSS: *n* = 472; and EMASPOT: *n* = 641). Analysis of missing data revealed comparatively low missing rates ranging from 3.1% (*n* = 44; life satisfaction) to 4.2% (*n* = 60; health status) in outcome variables and 0 to 7.5% (*n* = 107; MTS category) in independent variables.

In the final sample, sex was almost equally distributed with 50.9% of participants identifying as male and 49.1% as female (see Table 1). Mean age at baseline was 65.18 (SD: 16.72) years with a range between 18 and 98 years. In general, the sample comprised a large proportion of retired participants (58.0%), participants without migration background (77.1%) and of participants with at least three close social contacts (62.6%). Eighteen percent of participants were care dependent; almost 80% visited their GP at least once during the previous 6 months. However, 29.8% of participants reported to have visited the ED at least once during the previous 6 months and 24.6% reported having been hospitalized at least once in the respective period. Concerning their current ED presentation, 69.5% of participants were classified as urgent and 71.7% were hospitalized after ED treatment. Further sociodemographic, disease-specific and care-related characteristics of participants are reported in Table 1.

**Table 1:** Descriptive statistics of participants’ sociodemographic, disease-specific and care-related characteristics

Variable	n (%)
Study	
EMAAge	322 (22.4)
EMACROSS	472 (32.9)
EMASPOT	641 (44.7)
Sex	
Male	730 (50.9)
Female	705 (49.1)
Education level (CASMIN)^a^	
Primary level	432 (30.1)
Secondary level	549 (38.3)
Tertiary level	404 (28.2)
Employment status^b^	
Employed	414 (28.9)
Retired	833 (58.0)
Not (regularly) employed	103 (7.2)
Others	41 (2.9)
Migration background^c^	
Yes	298 (20.8)
No	1107 (77.1)
Number of close persons^d^	
None	58 (4.0)
1 or 2 persons	438 (30.5)
3 or more persons	899 (62.6)
Care dependency^e^	
Yes	260 (18.1)
No	1149 (80.1)
GP visit^1f^	
Yes	1132 (78.9)
No	268 (18.7)
ED visit^1g^	
Yes	428 (29.8)
No	953 (66.4)
Hospital stay^1h^	
Yes	353 (24.6)
No	1029 (71.7)
Case type^2i^	
Outpatient	454 (31.6)
Inpatient	981 (68.4)
MTS level^2j^	
Urgent (MTS level 1 through 3)	998 (69.5)
Non-urgent (MTS level 4 and 5)	330 (23.0)
Transportation to ED^2k^	
Walk-in	606 (42.2)
Non-urgent medically accompanied transport^3^	121 (8.4)
Emergency medical services	489 (34.1)
EMS with emergency physician	142 (9.9)

Note: *SD* Standard deviation, *CASMIN* Comparative Analysis of Social Mobility in Industrial Nations, *GP* General practitioner, *ED* Emergency department, *MTS* Manchester Triage System, *EMS* Emergency medical services; ^a^n = 50 missings; ^b^n = 44 missings; ^c^n = 30 missings; ^d^n = 40 missings; ^e^n = 26 missings; ^f^n = 35 missings; ^g^n = 54 missings; ^h^n = 53 missings; ^i^n = 60 missings; ^j^n = 107 missings; ^k^n = 77 missings; ^1^Question refers to use in the previous six months; ^2^Data extracted from patients’ electronic medical records; ^3^This transport form refers to the term “Krankentransport” in Germany, which describes the transportation of non-urgent, mostly chronically ill and mobility-impaired patients

Bivariate associations between independent variables and outcome variables were predominantly low (see Table 2). Moderate associations were observed between age and the following variables: sub-study (η^2^ = .514, *p* < .001), employment status (η^2^ = .764, *p* < .001), care dependency (η^2^ = .366, *p* < .001), case type (η^2^ = .460, *p* < .001) and transportation to the ED (η^2^ = .416, *p* < .001). Furthermore, case type and sub-study (*V* = .436, *p* < .001) as well as ED visit and hospital stay in the previous 6 months (*V* = .480, *p* < .001) were moderately correlated.

**Table 2:** Bivariate associations between participants’ sociodemographic, disease-specific and care-related characteristics and study outcomes

	1	2	3	4	5	6	7	8	9	10	11	12	13	14	15	16
1 Sub-study		.200**	.119**	.222**	.195**	.068*	.274**	.056	.070*	.098*	.436**	.287**	.335**	.514**	.188**	.140**
2 Sex	.200**		.091*	.111*	.013	.062	.107**	.070*	.018	.035	.053*	.058*	.123**	.065*	.015	.046
3 Education level	.119**	.091*		.226**	.121**	.138**	.249**	.107**	.064	.077*	.157**	.095*	.159**	.315**	.105*	.034
4 Employment status	.222**	.111*	.226**		.241**	.118**	.352**	.194**	.114*	.158**	.345**	.213**	.198**	.764**	.184**	.187**
5 Migration background	.195**	.013	.121**	.241**		.017	.079*	.058*	.019	.035	.217**	.162**	.154**	.284**	.053	.004
6 Number of close persons	.068*	.062	.138**	.118**	.017		.118**	.061	.051	.044	.027	.032	.086*	.089*	.111**	.182**
7 Care dependency	.274**	.107**	.249**	.352**	.079*	.118**		.092*	.134**	.182**	.187**	.075*	.289**	.366**	.224**	.136**
8 GP visit	.056	.070*	.107**	.194**	.058*	.061	.092*		.114**	.085*	.058*	.080*	.072	.183**	.042	.006
9 ED visit	.070*	.018	.064	.114*	.019	.051	.134**	.114**		.480**	.005	.001	.048	.057*	.133**	.080*
10 Hospital stay	.098*	.035	.077*	.158**	.035	.044	.182**	.085*	.480**		.104**	.041	.083*	.118**	.182**	.074*
11 Case type	.436**	.053*	.157**	.345**	.217**	.027	.187**	.058*	.005	.104**		.284**	.371**	.460**	.132**	.002
12 MTS level	.287**	.058*	.095*	.213**	.162**	.032	.075*	.080*	.001	.041	.284**		.192**	.293**	.047	.011
13 Transportation to ED	.335**	.123**	.159**	.198**	.154**	.086*	.289**	.072	.048	.083*	.371**	.192**		.416**	.089*	.084*
14 Age	.514**	.065*	.315**	.764**	.284**	.089*	.366**	.183**	.057*	.118**	.460**	.293**	.416**		−.089*	.043
15 Self-reported health	.188**	.015	.105*	.184**	.053	.111**	.224**	.042	.133**	.182**	.132**	.047	.089*	−.089*		.289**
16 Life satisfaction	.140**	.046	.034	.187**	.004	.182**	.136**	.006	.080*	.074*	.002	.011	.084*	.043	.289**	

Note: Numbers in the top row mirror the numbering of study variables in the first column. Associations between study variables can be read off the respective combination of information from the first column and top row. * α-level or p-level < .05; ** α-level or p-level < .001; MTS Manchester Triage System; Variable numbers 1 to 13 employ nominal scale level, while variable numbers 14 to 16 employ metric scale level; Statistical measures of associations: Cramer’s *V* for associations between nominal variables, Eta coefficient (*η*^*2*^) for associations between nominal and metric variables and, Pearson’s *r* for associations between metric variables

### Self-reported health

In our sample, mean ratings of self-reported health were 50.10 (SD: 23.62). Results of multilevel linear regression analyses revealed that better self-rated health was associated with affiliation to the EMASPOT sub-study (β = 4.35, *p* = .036; i.e., patients with cardiac symptoms) in comparison to EMAAge (see Table 3; i.e., patients with proximal femoral fractures). EMACROSS participants (i.e., patients with respiratory symptoms) reported lower health (β = − 5.72, *p* = .015) in comparison to EMAAge participants. Worse health status was associated with being in need of nursing care (β = − 7.52, *p* < .001) and not being (regularly) employed (β = − 8.54, *p* = .002) in comparison to those participants in employment. Female sex was associated with better health (β = 3.30, *p* = .014). Having had at least one hospital stay in the previous 6 months (β = − 4.67, *p* = .007) was associated with diminished self-reported health. Our multivariable model explained 18.9% of variance in self-reported health (conditional R^2^ = 0.189), whereby explained variance on the ED level amounted to 2.9% (ICC = 0.029).

**Table 3:** Estimates for fixed and random effects from multilevel linear regression analysis (random intercept model) for self-reported health as dependent variable and goodness-of-fit statistics

Fixed effects	Coefficient	95% CI	SE	*p*-value
Intercept	38.81	27.78; 51.77	6.31	<.001
Sex: Female	3.30	0.53; 5.76	1.35	.014
Study (reference category: EMAAge):				
EMACROSS	−5.72	−10.64; − 1.65	2.36	.015
EMASPOT	4.35	0.32; 8.28	2.07	.036
Education (reference category: Primary level)				
Secondary level	0.09	−2.93; 3.53	1.67	.958
Tertiary level	0.67	−2.52; 4.57	1.85	.717
Social contacts (reference category: None)				
1–2 persons	4.50	−2.33; 11.79	3.63	.215
3 or more persons	3.87	−2.80; 11.08	3.57	.278
Care dependency: Yes	−7.52	−11.49; −3.70	2.00	<.001
Migration background: Yes	2.06	−1.21; 5.43	1.71	.228
Employment status (reference category: Employed):				
Retired	−3.32	−7.59; 0.92	2.19	.130
Not (regularly) employed	−8.54	−14.05; −3.38	2.74	.002
Other	−3.99	−11.22; 3.63	3.82	.297
ED visit: Yes	−2.29	−5.48; 0.79	1.61	.156
Hospital stay: Yes	−4.67	−8.09; −1.35	1.73	.007
GP visit: Yes	0.88	−2.40; 4.33	1.73	.610
MTS level: Non-urgent	2.60	−1.01; 5.46	1.68	.121
Transportation to ED (reference category: Walk-in):				
Non-urgent medically accompanied transport	−0.54	−6.08; 4.18	2.65	.839
Emergency medical services	1.35	−1.82; 4.63	1.66	.417
EMS with emergency physician	2.90	−1.53; 7.37	2.29	.205
Age (in years)	−0.12	−0.27; 0.02	0.07	.093
Life satisfaction	2.22	1.68; 2.75	0.28	<.001
Random effects	Variance Component		SD	
Level-two variance: ED	6.74		2.60	
Level-one variance:	457.24		21.38	
Marginal R^2^ (fixed effects):	0.1769			
Conditional R^2^ (fixed and random effects):	0.1888			
ICC:	0.0286			

Note: *N* = 1096; Level 2: *n* = 8 emergency departments; *CI* Confidence interval, *SE* Standard error, *SD* Standard deviation, *ED* Emergency department, *GP* General practitioner, *ICC* Intraclass correlation coefficient, *MTS* Manchester Triage System, *EMS* Emergency medical services

### Life satisfaction

Mean life satisfaction in our sample amounted to 7.15 (SD: 2.50). Results of multilevel linear regression analyses revealed that higher life satisfaction was associated with affiliation to the EMASPOT sub-study (β = 0.53, *p* = .006; i.e., patients with cardiac symptoms) in comparison to EMAAge (see Table 4; i.e., patients with proximal femoral fractures). EMACROSS participants reported higher life satisfaction (β = 0.52, *p* = .021; i.e., patients with respiratory symptoms) in comparison to EMAAge participants. Lower life satisfaction was associated with being in need of nursing care (β = − 0.59, *p* = .003) and not being (regularly) employed (β = − 1.27, *p* < .001) in comparison to those participants in employment. Female sex was associated with lower life satisfaction (β = − 0.28, *p* = .036). Higher age (β = 0.02, *p* = .008) as well as having three or more close social contacts (β = 1.37, *p* < .001) was significantly related to higher life satisfaction. Self-reported health and life satisfaction were significantly associated, suggesting better self-perceived health with higher life satisfaction (β = 2.22, *p* < .001) and vice versa (β = 0.03, p < .001). Our multivariable model explained 15.3% of variance in life satisfaction (conditional R^2^ = 0.153), whereby explained variance on the ED level amounted to 0.6% (ICC = 0.006).

**Table 4:** Estimates for fixed and random effects from multilevel linear regression analysis (random intercept model) for life satisfaction as dependent variable and goodness-of-fit statistics

Fixed effects	Coefficient	95% CI	SE	*p*-value
Intercept	3.57	2.39; 4.84	0.62	<.001
Sex: Female	−0.28	−0.55; −0.02	0.14	.036
Study (reference category: EMAAge):				
EMACROSS	0.52	0.07; 0.96	0.23	.021
EMASPOT	0.53	0.15; 0.90	0.19	.006
Education (reference category: Primary level):				
Secondary level	−0.14	−0.47; 0.18	0.17	.412
Tertiary level	−0.21	−0.60; 0.13	0.18	.242
Social contacts (reference category:None):				
1–2 persons	0.72	−0.01; 1.45	0.38	.055
3 or more persons	1.37	0.65; 2.08	0.37	<.001
Care dependency: Yes	−0.59	−0.98; − 0.21	0.20	.003
Migration background: Yes	0.24	−0.10; 0.57	0.17	.170
Employment status (reference category: Employed):				
Retired	−0.07	−0.49; 0.36	0.22	.740
Not (regularly) employed	−1.27	−1.80; −0.73	0.27	<.001
Other	0.06	−0.71; 0.84	0.40	.879
ED visit: Yes	−0.16	−0.48; 0.16	0.16	.313
Hospital stay: Yes	−0.04	−0.37; 0.31	0.17	.840
Age (in years)	0.02	0.005; 0.03	0.01	.008
Self-reported health status	0.03	0.02; 0.03	< 0.01	<.001
Case type: Inpatient	0.02	−0.31; 0.33	0.16	.911
Random effects	Variance Component		SD	
Level-two variance: ED	0.02		0.14	
Level-one variance:	5.20		2.28	
Marginal R^2^ (fixed effects):	0.1501			
Conditional R^2^ (fixed and random effects):	0.1531			
ICC:	0.0058			

Note: *N* = 1245; Level 2: n = 8 emergency departments; *CI* Confidence interval, *SE* Standard error, *SD* Standard deviation, *ED* Emergency department, *ICC* Intraclass correlation coefficient

Sensitivity analyses excluding the EMAAge population (scenario B; *N* = 911; i.e., patients with proximal femoral fractures) indicated better self-reported health in the EMASPOT population (β = 9.44, p < .001; i.e., patients with cardiac symptoms) in comparison to EMACROSS (i.e., patients with respiratory symptoms). Other associations remained unchanged in their direction and statistical significance compared to the original sample (see Supplementary Table 1). Sensitivity analyses in scenario B (*N* = 1007) for life satisfaction was not feasible due to an error report for the statistical model indicating singular fit. Sensitivity analyses for self-reported health as an outcome in scenario C (exclusion of all participants under the age of 50; *N* = 944) revealed that retired participants reported significantly worse health (β = − 4.88, *p* = .041) than participants in employment. Other associations remained unchanged in their direction and statistical significance compared to the original sample (see Supplementary Table 2). Regarding life satisfaction as an outcome in scenario C (*N* = 1083), female sex (β = − 0.26, *p* = .075) was no longer significantly associated with satisfaction, although directions remained the same. Other associations remained unchanged in their direction and statistical significance compared to the original sample (see Supplementary Table 3).

## Discussion

This analysis of patient-reported outcomes from a cross-sectional data set of the multi-center study EMANET of ED patients in Germany’s capital Berlin indicated that self-reported health and life satisfaction in mostly older ED patients were associated with several sociodemographic and disease-related variables. Specifically, care dependency and unemployment significantly affected both diminished self-rated health and life satisfaction in ED patients. The following discussion incorporates separate reflections of concomitant factors of self-reported health and life satisfaction in ED patients by consulting prior research and nationally representative scores of both outcomes in the German general population.

### Self-reported health in ED patients

Self-reported health in our sample (mean: 50.1) was considerably lower compared to representative samples of German adults in general (mean: 77.3) and for specific age groups (means ranged from 85.3 for participants between 18 and 24 years and 60.5 for participants of 75 years and older) [38]. Rather low to medium ratings of patients’ health in the ED setting might be explained by at least two approaches: an acute deterioration in a patient’s health status leading to ED presentation or a long-term decline in self-perceived health leading to increased vulnerability. The former approach indicates that respective patients actually experience a significant measurable deterioration in their health status leading to presentation to an acute care facility. Our findings of poor self-reported health are in line with past research in different ED populations where health was measured with comparable instruments [17, 18]. Self-reported measures of health might thus constitute snapshots of actual health states with high sensitivity to changes in self-perceived health and potentially high variability of this measure over time. An ED visit would thus indicate a serious health event with immediate impact on patients’ subjective well-being [15]. Low to medium ratings of self-reported health in our sample on the other hand, lead to the assumption that our elderly multimorbid population already belonged to a high-risk population for adverse health outcomes and consistent diminished self-perceived health. Thus, specific acute and health-related events might not be the sole explanation for a drop in an otherwise good health of respective patients. Our finding that variables referring to the ED visit in question, like triage score and transportation to the ED, were not significantly associated with self-reported health in multivariable analysis might support this conjecture. Due to the cross-sectional nature of our study, these assumptions about temporal trajectories require consideration in future research where self-reported health is measured longitudinally, e.g., before, during and after an ED visit.

### Life satisfaction in ED patients

Life satisfaction in our study population (mean: 7.15, SD: 2.50) was comparable to research in representative samples of German adults in general (mean: 7.18, SD: 2.07) and for specific age groups (means ranged from 7.21 for participants between 18 and 35 years and 7.25 for participants of 65 years and older) [32]. Individual reactions to life events and certain circumstances were further found to be influenced by a person’s previous experiences, values and expectations [26]. Since life satisfaction in our sample of ED patients was comparable to ratings in representative population samples [26], our study results might indicate that life satisfaction indeed represents a rather stable construct, which is not highly affected by the current situation or mood of an individual. Satisfaction with one’s health is only one factor that individuals take into account when evaluating their general satisfaction with life [26]. In older multimorbid populations, research found that adaptation processes and/or coping methods are applied to handle chronic health conditions and accompanying functional limitations, thus altering the perception of personal restrictions and disease severity [26]. However, patients in poor health states might also “downplay the importance of their health when evaluating their global life satisfaction” ( [27] , p. 287) or feel obliged to positively evaluate their well-being in order to please close others [32]. Further research is needed to ascertain trajectories in this outcome concerning susceptibility to serious acute health events.

### Associated factors of self-reported health and life satisfaction

In a recent scoping review, predictors of self-reported health in older community-dwelling adults were found to be different sociodemographic factors, physical and mental health, health-related behavior and emotional factors [39]. Our research adds to existing studies by identifying sociodemographic, disease-specific and care-related factors associated with self-reported health specifically in the ED setting. Patients with higher life satisfaction showed better self-reported health consistent with previous research [22, 39]. Furthermore, negative associations between care dependency, unemployment, hospital stays in the previous 6 months and self-reported health supported previous findings [39]. Our results might indicate that underlying health problems in our sample of ED patients and accompanying limitations in functional abilities were highly correlated with our measures of care dependency and occupational status and were thus significantly associated with self-rated health. However, we found that patients with cardiac symptoms (i.e., EMASPOT study participants) reported significantly better health and life satisfaction than patients with respiratory symptoms (i.e., EMACROSS study participants) and those with proximal femoral fractures (i.e., EMAAge participants), even after controlling for sociodemographic factors. Thus, patients from distinct disease groups – according to the findings of this study – seem to differ in adaption processes and the perception or standards of ‘good’ health [7]. Perceived severity of symptoms leading to an ED consultation may also influence the rating of patient’s health status. Probably the impact of certain (chronic) medical conditions on functional limitations or other restraints in daily activities is visible in our results of self-rated health. ED patients with hip fractures who reported a diminished health status belong to a highly multimorbid geriatric group with multiple physical limitations and diminished health [40]. Similarly, patients with respiratory complaints were found to report a high burden of disease and severity of symptoms [39], thus rendering patients with cardiac symptoms the least susceptible to perceptions of poor health compared to ED patients with respiratory symptoms and those with hip fractures. Furthermore, age was not significantly associated with self-reported health, which is in line with an overall inconsistent state of research on this association [39]. Female sex was associated with better self-reported health in our sample which is in contrast with past research reporting significantly lower self-reported health in women than in men irrespective of age [41]. However, a recent review found that the evidence on this association in older adults is inconsistent [42]. Frequent ED users were more likely to report fair or poor health status than other ED users [43]. However, we did not find significant associations between ED use in the previous 6 months and self-rated health.

Finally, we ran multilevel analyses to adjust for potential effects on outcomes from differences regarding structural factors and the patient case-mix at our eight study sites. Results revealed only minimal importance of the adjustment of the respective study ED for the explanation of variance in self-reported health (between 2.9 and 4.4%) and specifically life satisfaction (between 0.4 and 0.6%). However, interestingly, excluding EMAAge participants and younger participants in sensitivity analyses was associated with a higher percentage of variance explained on the ED level for self-reported health, which might indicate slightly different age and case-mix distributions in ED populations between study sites.

### Implications

In previous research, self-reported health and life satisfaction were not only associated with mortality but also with favorable emotional factors and positive health-related behaviors, e.g., increased positive affect or physical activity, respectively [20, 39, 44]. If ED visits pose a serious health event, especially for older patients, additional interventions to strengthen subsequent use of beneficial health-related strategies and positive self-perceptions of ageing should be applied since both factors influence future health and life satisfaction in the elderly [6]. The discussion on the clinical use of measures of self-reported health and life satisfaction is pending. However, being able to identify especially vulnerable patients in the ED setting, e.g., by systematically enquiring PROs during patient’s ED stay or trustful patient-clinician interactions, might facilitate patient-centered care and prevent negative health outcomes. Thus, surveying information on PROs could be used to monitor patient progress in the individual patient-clinician interaction in the case of subsequent ED presentations [45]. The consideration of PROs and the psychosocial and emotional needs of patients at the onset and during an ED stay may increase health and well-being outcomes in vulnerable patient groups. Innovative approaches to cater to the psychosocial needs of patients in clinical environments are in demand. In order to improve patient experience in the ED, the use of additional personnel might be promising in addressing the social and personal needs of older ED patients who are at risk of adverse outcomes. This may include offering support for unaccompanied patients or to those with hearing, visual or cognitive impairments [11] or offering interdisciplinary support for patients with social needs [46].

### Limitations

Despite the usage of data from a multi-center study with a large sample size, our study is subject to different limitations. Due to the cross-sectional nature of this study, previous levels of self-reported health and life satisfaction in patients before the ED stay were unknown. Thus, any changes to baseline levels in both outcomes were not ascertained. Furthermore, we did not study the impact of other potentially relevant aspects, as they were outside the scope of the research network EMANet. Factors that might explain further variation in subjective well-being and health include coping abilities [26], use of health-related strategies [6], exposure to traumatic events [19] or further health determinants such as health literacy [27]. Our study was set in a high-income country which limits generalizability to other countries with less favorable conditions regarding household income or access to health services [27]. Biases, due to social desirability in the interview situation, might have positively skewed patient reports of health status and life satisfaction [47]. Furthermore, patients’ ability to remember and correctly report information, e.g., on the previous use of the healthcare system, might have been influenced by their acute and threatening health situation in the ED or by being surveyed after the ED stay, which applied to patients with proximal femoral fractures (i.e., EMAAge participants). However, we examined the potential latter bias by conducting sensitivity analyses excluding EMAAge participants in scenario B which did not show evidence for respective distortions. Finally, multilevel regression analysis models only explained a moderate amount of variance in our outcomes between 15% (life satisfaction) and 19% (self-reported health), which indicates that future research should account for further relevant variables. Furthermore, our multilevel analytic approach revealed minimal importance of the respective study ED for the explanation of variance in outcomes (between 0.6 and 2.9%).

## Conclusions

Self-reported health and life satisfaction were associated with different sociodemographic and disease-related variables in older ED patients. Specifically, care dependency and unemployment emerged as significant factors relating to worse self-reported health and lower life satisfaction. Being able to identify especially vulnerable patients in the ED setting might facilitate patient-centered care and prevent negative health outcomes. However, further longitudinal research needs to analyze trajectories in self-reported health and life satisfaction and suitable intervention possibilities in the ED setting.

## Supplementary Information

**Additional file 1: Table S1.** Inclusion criteria for the three EMANet sub-studies EMAAge, EMACROSS, and EMASPOT.

**Additional file 2: Table S2.** Scenario B estimates for fixed and random effects from multilevel linear regression analysis (random intercept model) for self-reported health as dependent variable and goodness-of-fit statistics.

**Additional file 3: Table S3.** Scenario C estimates for fixed and random effects from multilevel linear regression analysis (random intercept model) for self-reported health as dependent variable and goodness-of-fit statistics.

**Additional file 4: Table S4.** Scenario C estimates for fixed and random effects from multilevel linear regression analysis (random intercept model) for life satisfaction as dependent variable and goodness-of-fit statistics.

## Data Availability

Datasets are available from the corresponding author on reasonable request.

## Abbreviations

CASMIN: Comparative Analysis of Social Mobility in Industrial Nations
CI: Confidence interval
ED: Emergency department
EMAAge: Daily-life function and self-reported quality of life of geriatric patients before and after emergency treatment. Analysis of the status quo and potentials of health care improvements using the example of proximal femoral fracture
EMACROSS: Emergency and Acute Care for RespiratOry Diseases beyond Sectoral Separation
EMANet: Emergency and Acute Medicine Network for Health Care Research Berlin
EMASPOT: Systematic Screening for Comorbid Psychological Conditions in cardiac ACSC patients with multimorbidity in the ED
EMS: Emergency medical services
GP: General practitioner
ICC: Intraclass correlation coefficient
ICD-10: Tenth revision of the International Statistical Classification of Diseases and Related Health Problems
LS: Life satisfaction
MTS: Manchester Triage System
PRO: Patient-reported outcome
SD: Standard deviation
SRH: Self-rated health
VAS: Visual analogue scale

## Footnotes

1 The instrument used to monitor recruitment and sample quality in EMANet and initial results of the first 8 months of recruitment were described in the following German-language publication: Krobisch, V., Deutschbein, J., Möckel, M. et al. Empirische Versorgungsforschung in der Notfall- und Akutmedizin. Med Klin Intensivmed Notfmed 115, 125–133 (2020). 10.1007/s00063-018-0522-y.

2 The EMANet study group is currently working on a paper comprehensively delineating the representativity of the three samples.
