# Supplementary material for: Self-reported health and life satisfaction in older emergency department patients: sociodemographic, disease-related and care-specific associated factors
Source: BMC Public Health. 2021 Jul 21;21:1440. doi: 10.1186/s12889-021-11439-8 (PMC8296655; doi:10.1186/s12889-021-11439-8)
Supplement: Supplementary file 1 — Additional file 1: Table S1. Inclusion criteria for the three EMANet sub-studies EMAAge, EMACROSS, and EMASPOT. [file 12889_2021_11439_MOESM1_ESM.docx]

Supplementary Table 1: Inclusion criteria for the three EMANet sub-studies EMAAge, EMACROSS, and EMASPOT

| Inclusion criteria EMAAge:   - Age ≥ 18 years - Language speaking or reading ability in German, English, Arabic, or Turkish - Unique study participation in one of the three EMANet sub-studies - Appropriate physical and mental condition and ability to participate in a study   AND   - Presence of at least one of the following suspected ICD-10 diagnoses in the ED:   - S72.0 Fracture of neck of femur   - S72.1 Pertrochanteric fracture   - S72.2 Subtrochanteric fracture |
| --- |
| Inclusion criteria EMACROSS:   - Age ≥ 18 years - Out-patient treatment (up until September 2017)^a^ - Ability to give informed consent - Language speaking or reading ability in German, English, Arabic, or Turkish - Unique study participation in one of the three EMANet sub-studies - Appropriate physical and mental condition and ability to participate in a study   AND   - Presence of at least one of the following suspected ICD-10 diagnoses in the ED:   - J09 Influenza due to identified zoonotic or pandemic influenza virus   - J10 Influenza due to identified seasonal influenza virus   - J11 Influenza, virus not identified   - J12 Viral pneumonia, not elsewhere classified   - J13 Pneumonia due to Streptococcus pneumonia   - J14 Pneumonia due to Haemophilus influenza   - J15 Bacterial pneumonia, not elsewhere classified   - J16 Pneumonia due to other infectious organisms, not elsewhere classified   - J17 Pneumonia in diseases classified elsewhere   - J18 Pneumonia, organism unspecified   - J20 Acute bronchitis   - J21 Acute bronchiolitis   - J22 Unspecified acute lower respiratory infection   - J40 Bronchitis, not specified as acute or chronic   - J41 Simple and mucopurulent chronic bronchitis   - J42 Unspecified chronic bronchitis   - J43 Emphysema   - J44 Other chronic obstructive pulmonary disease   - J45 Asthma   - J46 Status asthmaticus   - J47 Bronchiectasis   OR   - Presence of at least one of the following main symptoms at ED presentation:   - Cough   - Shortness of breath / Dyspnea   - Expectoration   - Fever   - Common cold   - Sore throat   - Thoracic pain (in conjunction with respiratory complaints)   - Earache   - Fatigue   - Melalgia (in conjunction with respiratory complaints) |
| Inclusion criteria EMASPOT   - Age ≥ 50 years - Ability to give informed consent - Language speaking or reading ability in German, English, Arabic, or Turkish - Unique study participation in one of the three EMANet sub-studies - Appropriate physical and mental condition and ability to participate in a study   AND   - Presence of at least one of the following suspected ICD-10 diagnoses in the ED:   - I10 Essential (primary) hypertension   - I11.0 Hypertensive heart disease with (congestive) heart failure / I11.9 Hypertensive heart disease without (congestive) heart failure   - I20 Angina pectoris   - Excluded diagnosis of I21 Acute myocardial infarction   - I24.0 Coronary thrombosis not resulting in myocardial infarction / I24.8 Other forms of acute ischaemic heart disease / I24.9 Acute ischaemic heart disease, unspecified   - I47.1 Supraventricular tachycardia / I47.9 Paroxysmal tachycardia, unspecified   - I48.0 Paroxysmal atrial fibrillation / I48.2 Chronic atrial fibrillation / I48.9 Atrial fibrillation and atrial flutter, unspecified   - I49.5 Sick sinus syndrome / I49.8 Other specified cardiac arrhythmias / I49.9 Cardiac arrhythmia, unspecified   - I50 Heart failure   - J81 Pulmonary oedema   - R00.0 Tachycardia, unspecified / R00.1 Bradycardia, unspecified / R00.2 Palpitations / R00.8 Other and unspecified abnormalities of heart beat   - R07.3 Other chest pain / R07.4 Chest pain, unspecified   OR   - Presence of at least one of the following main symptoms at ED presentation:   - Chest pain   - Chest tightness   - Shortness of breath / Dyspnea   - Malaise   - Nausea   - Dizziness   - Weakness   - Fatigue   - Asthenia   - Problems with blood pressure   - Tachycardia   - Extrasystole   - Cardiac flutter   - Arrhythmia   - Swollen legs   - Weight gain   - Increase in abdominal girth |

Note: ICD-10 International Statistical Classification of Diseases and Related Health Problems 10th Revision; ^a^ The inclusion criterion “out-patient treatment” in EMACROSS was suspended during the recruitment process in September 2017 so that ED patients with and without hospital admission and other applicable criteria were eligible for participation from October 2017.
