# Supplementary material for: Self-reported health and life satisfaction in older emergency department patients: sociodemographic, disease-related and care-specific associated factors
Source: BMC Public Health. 2021 Jul 21;21:1440. doi: 10.1186/s12889-021-11439-8 (PMC8296655; doi:10.1186/s12889-021-11439-8)
Supplement: Supplementary file 2 — Additional file 2: Table S2. Scenario B estimates for fixed and random effects from multilevel linear regression analysis (random intercept model) for self-reported health as dependent variable and goodness-of-fit statistics. [file 12889_2021_11439_MOESM2_ESM.docx]

Supplementary Table 2: Scenario B estimates for fixed and random effects from multilevel linear regression analysis (random intercept model) for self-reported health as dependent variable and goodness-of-fit statistics

| Fixed effects | Coefficient | 95% CI | SE | p-value |
| --- | --- | --- | --- | --- |
| Intercept | 30.06 | 19.17; 41.93 | 5.99 | <.001 |
| Sex: Female | 3.33 | 0.34; 5.95 | 1.45 | .022 |
| Study (reference category: EMACROSS): EMASPOT | 9.44 | 6.41; 13.21 | 1.70 | <.001 |
| Education (reference category: Primary level):  Secondary level  Tertiary level | 0.92  0.74 | -2.42; 4.73  -2.88; 4.90 | 1.84  2.02 | .617  .714 |
| Social contacts (reference category: None):  1-2 persons  3 or more persons | 6.35  5.44 | -0.79; 14.19  -1.52; 13.19 | 3.86  3.79 | .100  .152 |
| Care dependency: Yes | -9.37 | -14.01; -4.95 | 2.33 | <.001 |
| Migration background: Yes | 1.60 | -1.85; 5.18 | 1.81 | .377 |
| Employment status (reference category: Employed):  Retired  Not (regularly) employed  Other | -2.70  -7.27  -2.31 | -7.16; 1.81  -13.04; -1.99  -9.64; 5.43 | 2.31  2.84  3.88 | .243  .011  .551 |
| ED visit: Yes | -1.79 | -5.11; 1.46 | 1.69 | .292 |
| Hospital stay: Yes | -5.36 | -9.06; -1.84 | 1.86 | .004 |
| GP visit: Yes | 0.16 | -3.42; 3.95 | 1.90 | .932 |
| MTS level: Non-urgent | 2.42 | -1.31; 5.55 | 1.74 | .165 |
| Transportation to ED (reference category: Walk-in):  Non-urgent medically accompanied transport  Emergency medical services  EMS with emergency physician | -1.15  1.61  3.76 | -7.42; 4.19  -1.64; 5.05  -0.99; 8.62 | 2.97  1.73  2.47 | .698  .350  .128 |
| Age (in years) | -0.11 | -0.26; 0.04 | 0.08 | .182 |
| Life satisfaction | 2.28 | 1.70; 2.88 | 0.30 | <.001 |

| Random effects | Variance Component | SD |
| --- | --- | --- |
| Level-two variance: ED | 7.76 | 2.79 |
| Level-one variance: | 441.75 | 21.02 |
| Marginal R^2^ (fixed effects): | 0.1890 | |
| Conditional R^2^ (fixed and random effects): | 0.2030 | |
| ICC: | 0.0438 | |

Note: N=911; Level 2: n=8 emergency departments; CI confidence interval; SE standard error; SD standard deviation; ED emergency department; GP general practitioner; MTS Manchester Triage System; EMS emergency medical services; ICC intraclass correlation coefficient.
