# Supplementary material for: Self-reported health and life satisfaction in older emergency department patients: sociodemographic, disease-related and care-specific associated factors
Source: BMC Public Health. 2021 Jul 21;21:1440. doi: 10.1186/s12889-021-11439-8 (PMC8296655; doi:10.1186/s12889-021-11439-8)
Supplement: Supplementary file 3 — Additional file 3: Table S3. Scenario C estimates for fixed and random effects from multilevel linear regression analysis (random intercept model) for self-reported health as dependent variable and goodness-of-fit statistics. [file 12889_2021_11439_MOESM3_ESM.docx]

Supplementary Table 3: Scenario C estimates for fixed and random effects from multilevel linear regression analysis (random intercept model) for self-reported health as dependent variable and goodness-of-fit statistics

| Fixed effects | Coefficient | 95% CI | SE | p-value |
| --- | --- | --- | --- | --- |
| Intercept | 33.27 | 18.25; 48.59 | 7.92 | <.001 |
| Sex: Female | 3.68 | 0.73; 6.36 | 1.45 | .011 |
| Study (reference category: EMAAge):  EMACROSS  EMASPOT | -5.76  4.81 | -10.81; -1.61  0.79; 9.03 | 2.43  2.16 | .018  .026 |
| Education (reference category: Primary level):  Secondary level  Tertiary level | 0.67  1.61 | -2.45; 4.35  -1.64; 5.82 | 1.76  1.96 | .702  .410 |
| Social contacts (reference category: None):  1-2 persons  3 or more persons | 3.00  2.16 | -4.02; 10.64  -4.71; 9.69 | 3.78  3.71 | .427  .561 |
| Care dependency: Yes | -7.80 | -11.79; -3.94 | 2.02 | <.001 |
| Migration background: Yes | 2.43 | -1.21; 6.34 | 1.95 | .212 |
| Employment status (reference category: Employed):  Retired  Not (regularly) employed  Other | -4.88  -8.64  -9.07 | -9.60; -0.32  -14.66; -2.72  -20.88; 3.03 | 2.39  3.08  6.16 | .041  .005  .141 |
| ED visit: Yes | -2.43 | -5.97; 0.85 | 1.76 | .167 |
| Hospital stay: Yes | -4.45 | -8.06; -0.90 | 1.85 | .016 |
| GP visit: Yes | 0.36 | -3.17; 4.32 | 1.93 | .851 |
| MTS level: Non-urgent | 2.59 | -1.34; 5.87 | 1.90 | .174 |
| Transportation to ED (reference category: Walk-in):  Non-urgent medically accompanied transport  Emergency medical services  EMS with emergency physician | 0.10  2.11  3.85 | -5.62; 4.90  -1.19; 5.52  -0.66; 8.53 | 2.72  1.73  2.36 | .971  .224  .104 |
| Age (in years) | -0.02 | -0.22; 0.18 | 0.10 | .853 |
| Life satisfaction | 2.22 | 1.63; 2.78 | 0.29 | <.001 |

| Random effects | Variance Component | SD |
| --- | --- | --- |
| Level-two variance: ED | 7.69 | 2.77 |
| Level-one variance: | 452.34 | 21.27 |
| Marginal R^2^ (fixed effects): | 0.1920 | |
| Conditional R^2^ (fixed and random effects): | 0.2055 | |
| ICC: | 0.0414 | |

Note: N=944; Level 2: n=8 emergency departments; CI: confidence interval; SE standard error; SD standard deviation; ED emergency department; GP general practitioner; MTS Manchester Triage System; EMS emergency medical services; ICC intraclass correlation coefficient.
