# Supplementary material for: Self-reported health and life satisfaction in older emergency department patients: sociodemographic, disease-related and care-specific associated factors
Source: BMC Public Health. 2021 Jul 21;21:1440. doi: 10.1186/s12889-021-11439-8 (PMC8296655; doi:10.1186/s12889-021-11439-8)
Supplement: Supplementary file 4 — Additional file 4: Table S4. Scenario C estimates for fixed and random effects from multilevel linear regression analysis (random intercept model) for life satisfaction as dependent variable and goodness-of-fit statistics. [file 12889_2021_11439_MOESM4_ESM.docx]

Supplementary Table 4: Scenario C estimates for fixed and random effects from multilevel linear regression analysis (random intercept model) for life satisfaction as dependent variable and goodness-of-fit statistics

| Fixed effects | Coefficient | 95% CI | SE | p-value |
| --- | --- | --- | --- | --- |
| Intercept | 2.62 | 1.15; 4.21 | 0.78 | <.001 |
| Sex: Female | -0.26 | -0.54; 0.02 | 0.15 | .075 |
| Study (reference category: EMAAge):  EMACROSS  EMASPOT | 0.57  0.63 | 0.11; 1.02  0.24; 1.02 | 0.23  0.20 | .015  .002 |
| Education (reference category: Primary level):  Secondary level  Tertiary level | -0.13  -0.28 | -0.48; 0.20  -0.68; 0.08 | 0.18  0.19 | .448  .148 |
| Social contacts (reference category: None):  1-2 persons  3 or more persons | 0.73  1.33 | -0.03; 1.49  0.58; 2.07 | 0.39  0.38 | .064  <.001 |
| Care dependency: Yes | -0.68 | -1.07; -0.29 | 0.20 | <.001 |
| Migration background: Yes | 0.15 | -0.23; 0.52 | 0.19 | .451 |
| Employment status (reference category: Employed):  Retired  Not (regularly) employed  Other | -0.21  -1.08  -0.30 | -0.67; 0.26  -1.70; -0.48  -1.59; 0.98 | 0.24  0.31  0.66 | .372  <.001  .644 |
| ED visit: Yes | -0.28 | -0.62; 0.07 | 0.18 | .113 |
| Hospital stay: Yes | 0.15 | -0.21; 0.52 | 0.19 | .432 |
| Age (in years) | 0.03 | 0.01; 0.05 | 0.01 | .001 |
| Self-reported health status | 0.02 | 0.02; 0.03 | 0.00 | <.001 |
| Case type: Inpatient | 0.04 | -0.31; 0.38 | 0.18 | .804 |

| Random effects | Variance Component | SD |
| --- | --- | --- |
| Level-two variance: ED | 0.02 | 0.13 |
| Level-one variance: | 5.23 | 2.29 |
| Marginal R^2^ (fixed effects): | 0.1551 | |
| Conditional R^2^ (fixed and random effects): | 0.1579 | |
| ICC: | 0.0036 | |

Note: N=1,083; Level 2: n=8 emergency departments; CI confidence interval; SE standard error; SD standard deviation; ED emergency department; ICC intraclass correlation coefficient.
